# The use of imageless navigation to quantify cutting error in total knee arthroplasty

**DOI:** 10.1186/s43019-021-00125-z

**Published:** 2021-12-04

**Authors:** Ran Schwarzkopf, Morteza Meftah, Scott E. Marwin, Michelle A. Zabat, Jeffrey M. Muir, Iain R. Lamb

**Affiliations:** 1 Department of Orthopaedic Surgery, NYU Langone Health, NYU Langone Orthopedic Hospital, 301 East 17th Street, New York, NY 10003 USA; 2 Intellijoint Surgical, Kitchener, ON Canada

**Keywords:** Knee, Osteoarthritis, Arthroplasty, Computer-assisted navigation, Saw blade deflection, Navigated TKA

## Abstract

**Purpose:**

Navigated total knee arthroplasty (TKA) improves implant alignment by providing feedback on resection parameters based on femoral and tibial cutting guide positions. However, saw blade thickness, deflection, and cutting guide motion may lead to final bone cuts differing from planned resections, potentially contributing to suboptimal component alignment. We used an imageless navigation device to intraoperatively quantify the magnitude of error between planned and actual resections, hypothesizing final bone cuts will differ from planned alignment.

**Materials and methods:**

A retrospective study including 60 consecutive patients undergoing primary TKA using a novel imageless navigation device was conducted. Device measurements of resection parameters were obtained via attachment of optical trackers to femoral and tibial cutting guides prior to resection. Following resection, optical trackers were placed directly on the bone cut surface and measurements were recorded. Cutting guide and bone resection measurements of both femoral and tibial varus/valgus, femoral flexion, tibial slope angles, and both femoral and tibial medial and lateral resection depths were compared using a Student's *t*-test.

**Results:**

Femoral cutting guide position differed from the actual cut by an average 0.6 ± 0.5° (*p* = 0.85) in the varus/valgus angle and 1.0 ± 1.0° (*p* = 0.003) in the flexion/extension angle. The difference between planned and actual cut measurements for medial and lateral femoral resection depth was 1.1 ± 1.1 mm (*p* = 0.32) and 1.2 ± 1.0 mm (*p* = 0.067), respectively. Planned cut measurements based on tibial guide position differed from the actual cut by an average of 0.9 ± 0.8° (*p* = 0.63) in the varus/valgus angle and 1.1 ± 1.0° (*p* = 0.95) in slope angle. Measurement of medial and lateral tibial resection depth differed by an average of 0.1 ± 1.8 mm (*p* = 0.78) and 0.2 ± 2.1 mm (*p* = 0.85), respectively.

**Conclusions:**

Significant discrepancies between planned and actual femoral bone resection were demonstrated for flexion/extension angle, likely the result of cutting error. Our data highlights the importance of cut verification postresection to confirm planned resections are achieved, and suggests imageless navigation may be a source of feedback that would allow surgeons to intraoperatively adjust resections to achieve optimal implant alignment.

## Introduction

Total knee arthroplasty (TKA) is one of the most common and effective surgical interventions for end-stage osteoarthritis of the knee joint [1, 2]. While advances in orthopedic technology have improved postsurgical outcomes, implant malalignment remains a predominate issue, with unsatisfactory prosthesis positioning reported in up to 30% of primary TKA procedures [3–6]. The well-documented association between malalignment, poor patient outcomes, and lower rates of implant survivability underscores the importance of optimizing component alignment [7–9]. Consequently, efforts to lessen both the prevalence and magnitude of alignment errors help mitigate associated complications, increasing the likelihood of long-term postoperative success and favorable patient outcomes [4, 10].

While the causes of malalignment are multifactorial, cutting errors associated with femoral and tibial resection are known to play a significant role [11, 12]. Bone cuts typically rely on an oscillating bone saw, the blade of which is subject to deflection, which may lead to movement of the cutting guide. This deflection has been shown to contribute to deviations from the planned plane of resection and, ultimately, to errors in component placement [11–13]. Cutting errors have been reported to cause deviations in component position of up to 4° in the coronal plane [11, 14] and 10° in the sagittal plane [11, 15], a magnitude of error that exceeds the ideal clinical range of ± 3° from the mechanical axis of the joint and can contribute to limb malalignment [9–11, 16–18]. Malalignment leads to uneven load distribution [6, 19] and suboptimal restoration of joint biomechanics [4, 5], both of which can adversely affect long-term procedural success, significantly elevate the rate of prosthesis failure, and increase the likelihood of future revision surgery [3, 5, 9, 20]. Furthermore, resection errors can result in uneven bone surfaces, causing gaps between bone and implant that have deleterious effects on alignment, implant adhesion in cemented cases, and bone ingrowth in uncemented implants [11, 12, 21]. Given the adverse impact of resection error on alignment, a better understanding of the magnitude and prevalence of this error is critical to mitigating it.

The integration of computer-assisted navigation systems (CAS) in TKA procedures has grown in popularity due to their ability to improve accuracy of component placement by providing real-time feedback on resection parameters based on tibial and femoral cutting guides [22]. This helps facilitate alignment to ensure optimal cut parameters are achieved, leading to an improvement in both component and limb alignment compared with conventional methods [23]. Additionally, CAS offers the ability to verify bone cuts intraoperatively postresection, allowing surgeons to make adjustments and modifications prior to implant placement that may optimize alignment. We utilized these features in a novel CAS system to compare planned and actual cut measurements for the femur and tibia in a cohort of patients undergoing primary TKA with computer navigation assistance. The difference between the planned and actual cuts provides an indication of the error associated with bone resection during primary TKA procedures. We hypothesized that final bone cuts will minimally differ from planned alignment.

## Materials and methods

### Study design and patient population

We conducted a retrospective review of patients who underwent a primary TKA with the assistance of imageless computer navigation between October 2019 and December 2019 at a single medical center. All procedures were performed by one of three senior fellowship-trained and board-certified surgeons specialized in total joint arthroplasty. Inclusion criteria required successful positioning of femoral and/or tibial cutting guides using imageless navigation and intraoperative measurement of pre- and postresection cuts by the imageless computer navigation device. Procedures in which the navigation device was removed prior to the recording of key measurements, or the measurements were not recorded or obtained from the device were excluded from analysis. This study received Institutional Review Board (IRB) approval from the institutional ethics board prior to data collection.

### Surgical technique

**Fig. 1:**
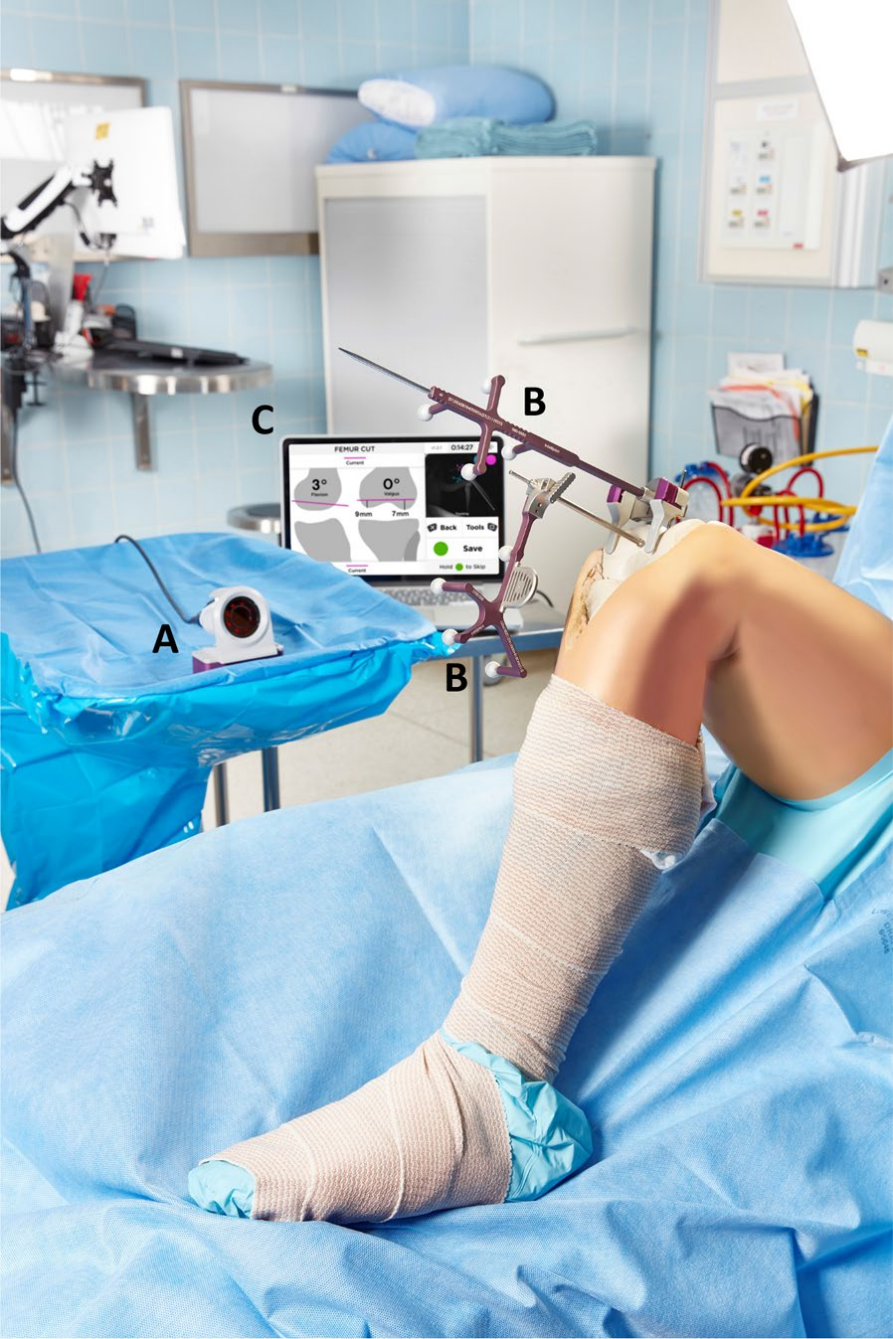
During use, the camera (**A**) detects movement of the trackers (**B**) within its field of view and relays information to the computer workstation (**C**), which sits outside of the sterile field. The workstation displays intraoperative data in real time and is controlled by the surgeon using buttons on the camera or by an assistant using the keyboard

**Fig. 2:**
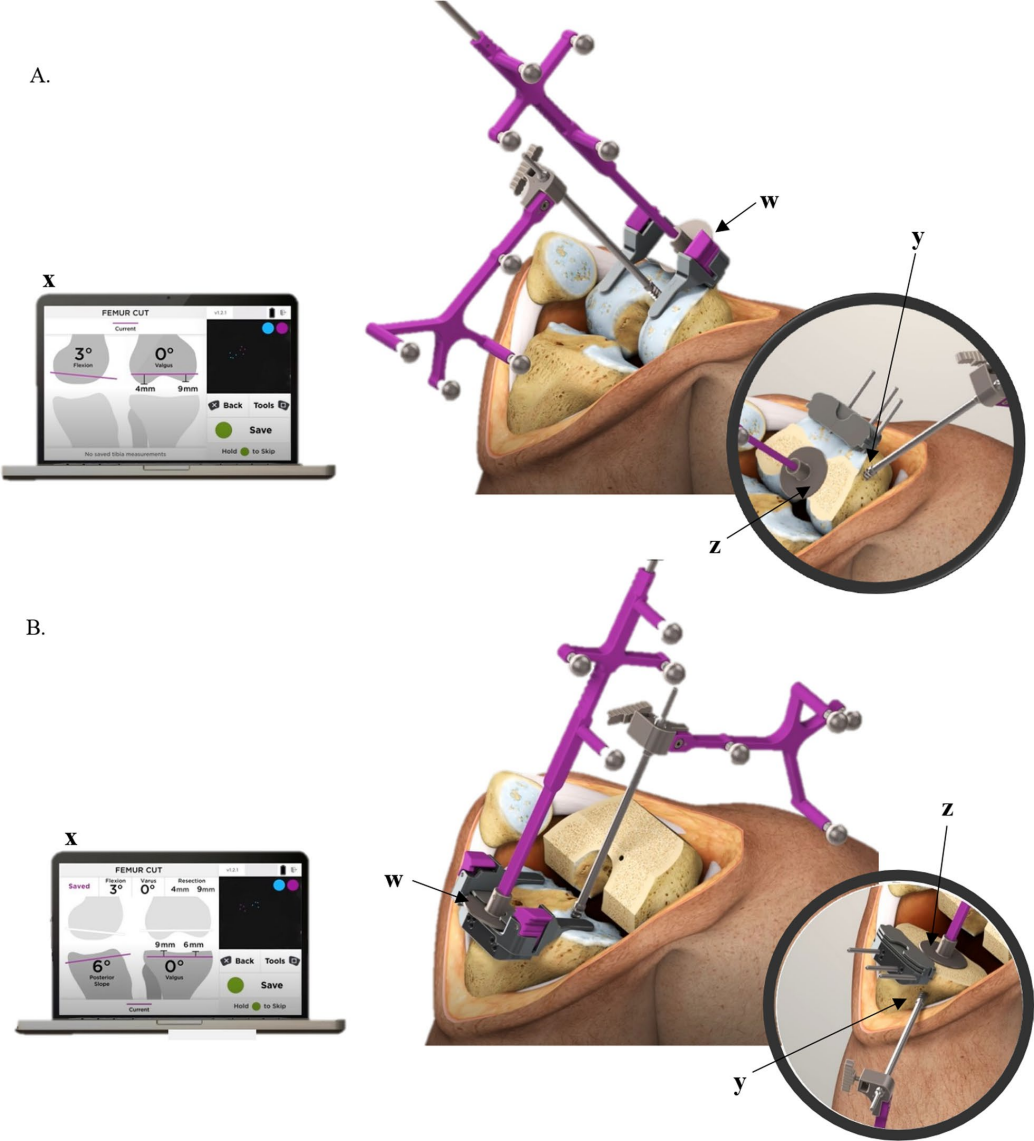
Imageless computer navigation device. **A** With optical probe tracker slotted into the femoral cutting guide (*w*) changes in guide position are detected by the camera (not pictured) and the impact on planned cut parameters are displayed on the workstation (*x*), in real time. Insert shows that with extraarticular installation of the bone screw (*y*), the probe tracker can be placed on femur postresection (*z*) and bone cut parameters are displayed on the workstation in real time. **B** With optical probe tracker slotted into the tibial cutting guide (*w*) changes in guide position are detected by the camera (not pictured) and the impact on planned cut parameters are displayed on the workstation (*x*) in real time. Insert shows that with extraarticular installation of the bone screw (*y*), the probe tracker can be placed on tibia postresection (*z*) and bone cut parameters are displayed on the workstation in real time

All procedures were performed via the medial parapatellar approach and used imageless computer navigation (Intellijoint KNEE™, Intellijoint Surgical, Kitchener, ON). Given this navigation device accommodates either a femur- or tibia-first workflow, the TKA procedures included in this study represent both workflows. The device is composed of a camera, optical trackers, and a computer workstation that sits outside of the sterile field and is controlled by the surgeon via buttons on the camera (Fig. 1). The workflow for tibial cuts is as follows: after the primary incision and exposure of the femur and tibia, a bone screw is drilled into the tibia and the optical bone tracker is attached. The bone screw may be inserted either on the articular or extraarticular surface of the proximal tibia; however, for cut verification, extraarticular placement of the screw is required. The tibia is registered using the optical probe tracker by defining the medial and lateral malleoli, the tibial center, the anteroposterior (AP) axis, and the medial and lateral tibial plateaus. The optical probe tracker is then attached to the cutting guide, providing real-time feedback of the prospective cut measurements. Cutting guide position can be adjusted to the desired orientation and then secured in place. Postresection, the tracker is placed on the cut surface and the bone cut is measured by the navigation device (Fig. 2).

The workflow for femoral cuts is similar to that for tibial cuts: a bone screw is drilled into the femur and the optical bone tracker is attached. Again, while the bone screw can be inserted either on the articular or extraarticular surfaces of the distal femur, cut verification requires extraarticular positioning of the screw. The femur is registered by rotating the hip joint through a range of motion to capture the hip center of rotation. The optical probe tracker is used to define the femur center, anterior–posterior femoral line, and the lateral and medial femoral condyles. As with the tibia, the optical probe tracker is attached to the cutting guide, providing real-time feedback to help position the cutting guide, which is secured with pins once the desired orientation is achieved. Postresection, the tracker is placed on the cut surface and the bone cut is measured by the navigation device (Fig. 2).

All resections were performed with an oscillating saw with a 1.27 mm saw blade. Multiple models of cutting guides were used; however, all were closed-slot guides designed for use with a 1.35 mm saw blade.

### Outcome measures

Patient demographics at the time of surgery [age, sex, and body mass index (BMI)] were collected. Cut parameters were measured by the navigation device for the femur and tibia prior to and immediately following each cut, and included varus/valgus angle, flexion/extension, posterior slope, and resection depth. The primary analysis was a comparison of pinned cutting guide positions (planned resections, based on preoperative evaluation of individual patient deformity) to postresection measurements (actual resections) of the tibia and femur cut surface.

### Statistical analysis

Data is expressed as mean ± standard deviation (SD). Paired Student’s *t*-tests were used to determine if there were any significant differences between mean pre- and postresection measurements. Alpha was set a priori at 0.05 for all statistical comparisons. All statistical analyses and calculations were performed using Microsoft Excel (Microsoft Corporation, Redmond, WA, USA).

## Results

### Study population

A total of 60 consecutive patients were identified for this study. Femur (*n* = 13) or tibia (*n* = 10) measurements were excluded from this study if the cutting guide or postresection cut measurement were not collected. Therefore, measurements of 47 femoral and 50 tibial resections were included in this study. No instances of loose cutting guides were reported.

Demographic data of patients included in the study is outlined in Table 1.

**Table.1:** Patient demographic data (*N* = 60)

Demographic data	
Age, mean (SD)	64.7 (10.0)
< 50	5
50–59	13
60–69	18
70+	20
Not reported	4
Sex	
Male	46
Female	12
Not reported	
BMI (*n* = 56), mean (SD)	33.5 (5.8)

### Femoral measurements

For the femur, the mean planned varus/valgus cut based on cutting guide orientation was 0.32 ± 1.54°, while the mean actual cut was 0.04 ± 1.54° (*p* = 0.85, Table 2). The mean absolute difference between planned and actual cuts was 0.64 ± 0.52° in the varus/valgus plane (Table 3). The mean planned femoral flexion/extension cut was measured at 4.04 ± 1.84° of flexion, while the actual cut was measured at 3.45 ± 1.67° of flexion following resection (Table 2), with a mean absolute difference of 1.03 ± 0.96° (Table 3). The difference between planned and actual resection in the flexion/extension plane was statistically significant (*p* = 0.003). The mean planned medial and lateral resection cuts were 11.15 ± 2.97 mm and 9.61 ± 2.97 mm, respectively, while measurements following resection were 10.93 ± 2.74 mm (*p* = 0.32) and 9.18 ± 3.51 mm (*p* = 0.067, Table 2), with mean absolute differences of 1.05 ± 1.10 mm and 1.24 ± 1.04 mm, respectively (Table 3).

**Table.2:** Average cut parameters for femoral and tibial resection

	Varus/valgus (°)			Slope (°)		
	Planned	Actual	*p*-Value	Plan	Actual	*p*-Value
Femur	0.32 ± 1.54	0.04 ± 1.54	0.85	4.04 ± 1.84	3.45 ± 1.67	0.003
Tibia	0.47 ± 1.26	0.54 ± 1.23	0.63	6.27 ± 2.07	6.28 ± 2.63	0.95

	Medial resection (mm)			Lateral resection (mm)		
	Planned	Actual	*p*-Value	Planned	Actual	*p*-Value
Femur	11.15 ± 2.97	10.93 ± 2.74	0.32	9.61 ± 2.97	9.18 ± 3.51	0.067
Tibia	6.34 ± 2.95	6.39 ± 2.12	0.78	8.53 ± 2.93	8.59 ± 2.66	0.85

**Table.3:** Average difference between planned and actual resection measurements for femoral and tibial resection

	Varus/valgus (°)	Slope (°)	Medial resection (mm)	Lateral resection (mm)
Femur	0.64 ± 0.52	1.03 ± 0.96	1.05 ± 1.10	1.24 ± 1.04
Tibia	0.91 ± 0.79	1.10 ± 1.00	0.10 ± 1.79	0.16 ± 2.09

**Fig. 3:**
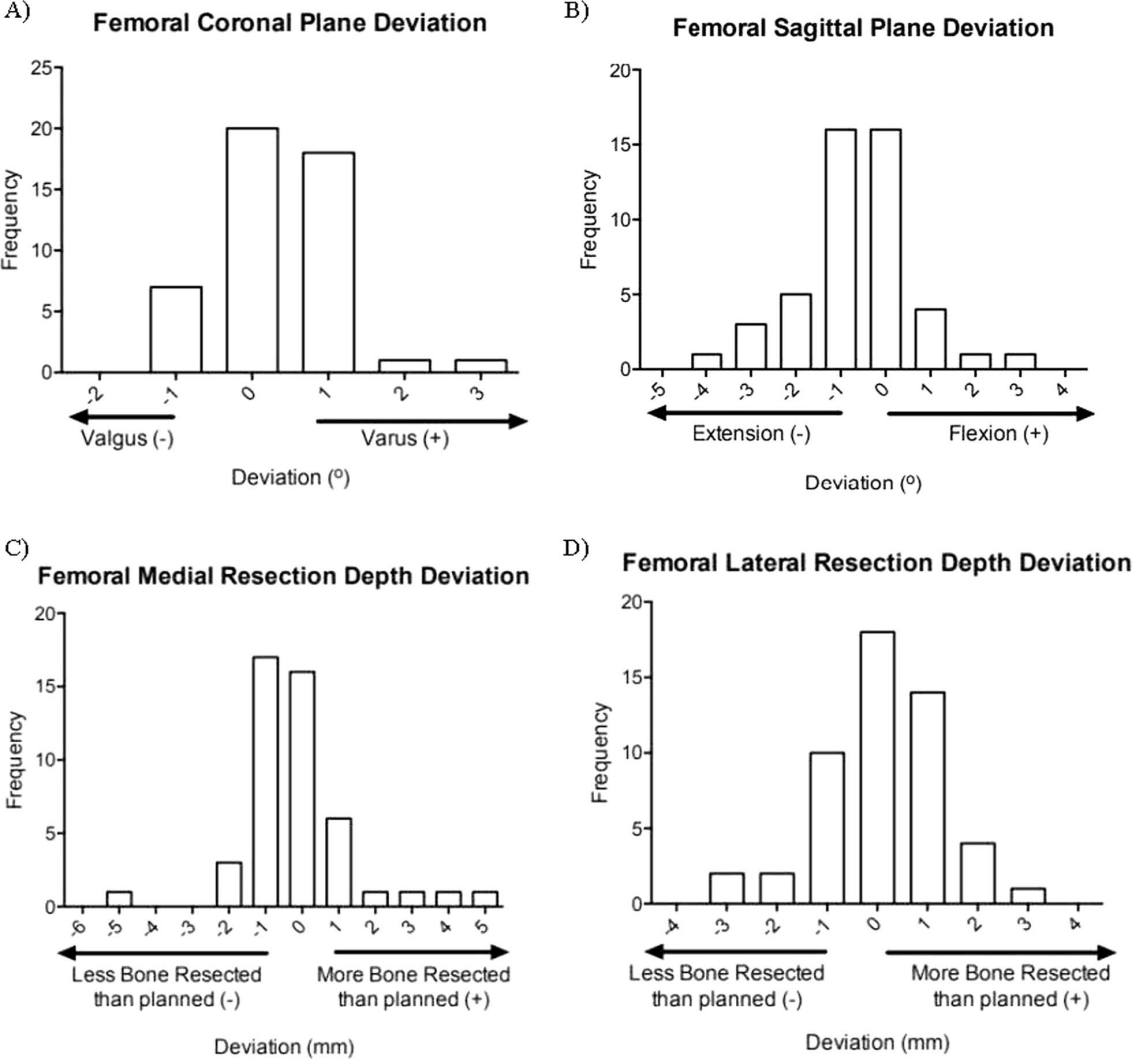
Distribution of deviation between planned and actual femoral resection in the **A** varus/valgus, **B** flexion/extension, **C** medial resection, and **D** lateral resection planes

All differences between planned and actual resection in the varus/valgus plane were within 3°, while 94% (44/47) were within 3° for flexion/extension. Ninety-four percent (44/47) of measurements for medial and 96% (45/47) of lateral cutting guide deviations were within 3 mm (Fig. 3) [24]. The full range of data for these femoral measurements can also be seen in Fig. 2.

### Tibial measurements

For the tibia, the mean planned valgus/varus cut based on cutting guide orientation was 0.47 ± 1.26° varus, while the mean actual cut was 0.54 ± 1.23° (*p* = 0.63, Table 2). The mean absolute difference was 0.91 ± 0.79° in the varus/valgus plane (Table 3). Mean planned resection slope was 6.27 ± 2.07° versus a mean actual cut slope of 6.28 ± 2.63° following resection (*p* = 0.95, Table 2), with a mean absolute difference of 1.10 ± 1.00° (Table 3). Mean preresection measurements of planned medial and lateral resection cuts were 6.34 ± 2.95 mm and 8.53 ± 2.93 mm, respectively, while measurements following resection were 6.39 ± 2.12 mm (*p* = 0.78) and 8.59 ± 2.66 mm (*p* = 0.85, Table 2), with a mean average difference of 0.10 ± 1.79 mm and 0.16 ± 2.09 mm (Table 3). There were no significant differences between the planned resection parameters and actual resection.

**Fig. 4:**
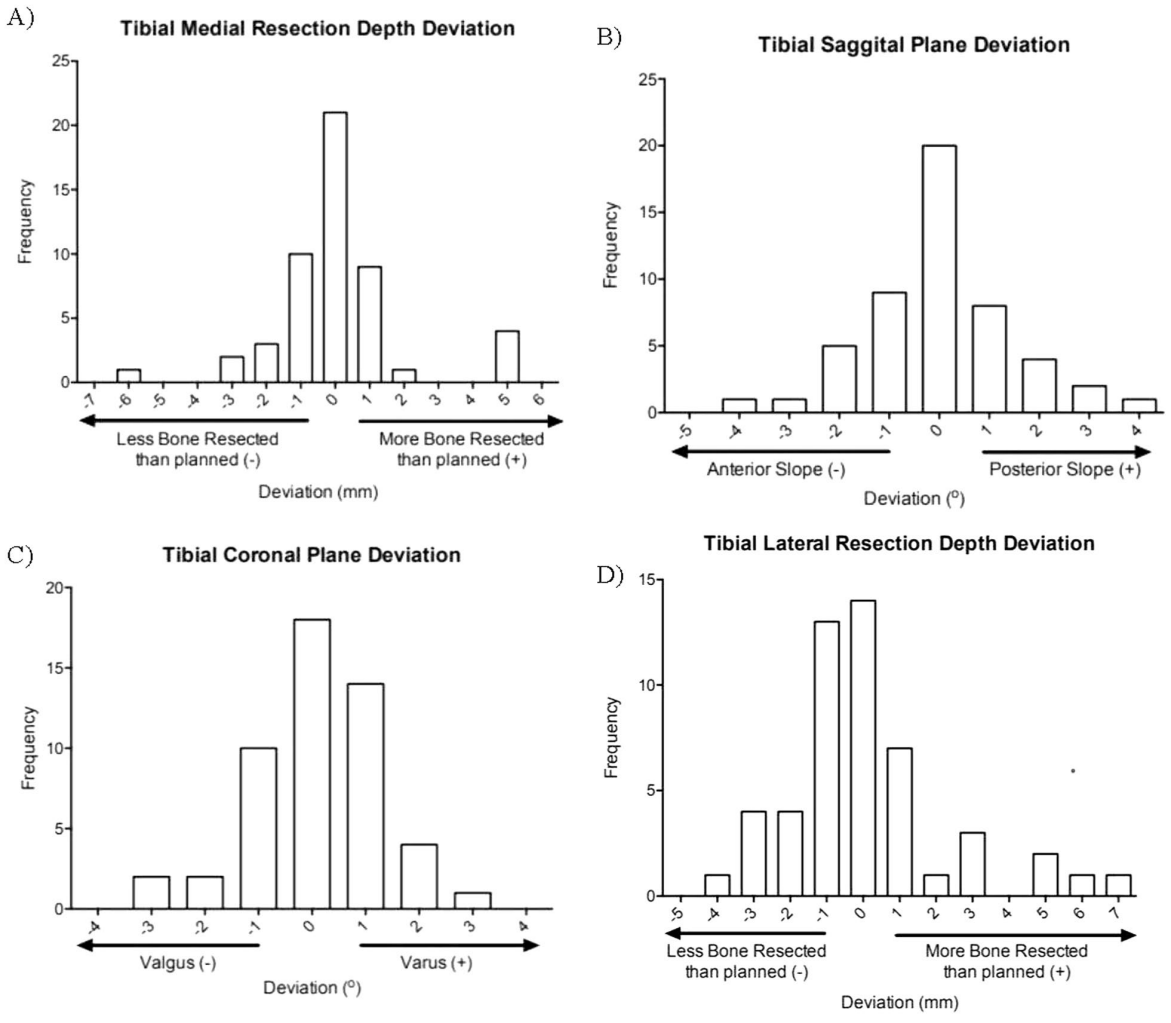
Distribution of deviation between planned and actual tibial resection in the **A** varus/valgus, **B** flexion/extension, **C** medial resection, and **D** lateral resection planes

Ninety-six percent (48/50) of differences between planned and actual resection in the varus/valgus plane were within 3°, and 96% (48/50) were within 3° for flexion/extension. Ninety-two percent (46/50) of measurements for medial and 90% (45/50) of lateral cutting guide deviations were within 3 mm (Fig. 4). The full range of data for these tibial measurements can also be seen in Fig. 3.

## Discussion

In primary TKA procedures, cutting errors during tibial and femoral resection have been shown to adversely impact component alignment [11–13]. Using an imageless computer navigation device, we quantified the extent to which planned and actual resection parameters differed, providing an indication of the magnitude of error associated with bone resection during primary TKA. We observed a statistically significant difference between planned and actual resection in the sagittal plane of the femur, and a difference between planned and achieved lateral femoral resection that trended toward significance. The data indicates that there is potentially important error associated with femoral and tibial cuts in TKA which may, in part, contribute to component and limb malalignment; additionally, this error can be assessed intraoperatively by imageless computer navigation.

We noted differences of 1.03° and 1.10° between planned and actual cuts in the femur and tibia, respectively, which mirror the results of similar studies. Chua et al. [25] noted errors of up to 1° in both tibial and femoral cuts, and determined these errors were potential causes of component malalignment noted on postoperative imaging. Other studies have identified alternative sources of component alignment error, including one study [26] that implicated cementing of components in place as a causal source of error of between 1° and 3°. Together, these findings suggest there are multiple sources of alignment error associated with resection cuts in TKA, which cumulatively push the error close to the threshold of ± 3° that is generally accepted as the maximal tolerance for postoperative alignment [9–11, 16–18]. Like other studies, we noted statistically significant differences in cuts in the femoral sagittal cuts [25]. Errors in the sagittal plane, especially in the femoral cut, have been reported to have an adverse impact on joint kinematics, range of motion, and component sizing, all of which adversely affect knee joint function [27]. Sagittal malpositioning of the femoral component has also been linked to inferior component survival and elevated risk for flexion contracture [7]. Tibial cut errors, meanwhile, threaten the accuracy of gap balancing and, therefore, the biomechanical stability of the new joint [28].

Drivers behind the greater frequency of sagittal plane cut errors are not fully known; however, one possibility is that sagittal plane resection may be more sensitive to differences in procedural factors. Plaskos et al. investigated these aspects, finding that the greater frequency of sagittal plane cut errors is influenced by surgeon experience, surgical instrumentation including cutting guide type, and cutting guide movement, all of which have been shown to impact the magnitude of resection error [11]. Deflection of the blade in the cutting guide represents another possible explanation for the observed differences. Several studies found that deflection or bending of the saw blade, especially when cutting through sclerotic bone, may lead to cutting error [29]. Poor bone mineral density in osteoporotic patients has also been suggested as a driver of poor tibial component position in CAS-assisted TKA, resulting from cutting guide pin motion during resection [30]. The use of slotted cutting guides or thicker blades have been suggested as potential solutions for these problems [11]; however, they do not completely eliminate the error associated with these cuts. In our study, we controlled for the hypothesized causal factors of surgeon experience, surgical instrumentation, and saw blade deflection by including only high-volume, experienced surgeons consistent in the use of 1.27 mm saw blades and 1.35 mm slotted cutting guides. However, we still observed differences between planned and actual cuts, suggesting that there are additional factors that contribute to the observed error that remain to be fully elucidated.

The use of computer-assisted navigation to confirm resection angles may provide an additional important cross check against inaccurate cuts. Previous studies have used navigation to monitor the orientation of cutting guides prior to actual cuts [25, 26], but the deflection that occurs during the cutting process warrants assessment of the actual cut angle itself prior to final component implantation. Further, the feedback provided by navigation devices may better characterize the impact that changes in surgical approach or equipment have on resection error, which could lead to alterations in the surgical workflow to help minimize cutting errors. For instance, the ability to verify and perform postresection adjustments may alter current approaches to preserving bone stock, which has been shown to decrease the risk of aseptic loosening, improve outcomes when the knee is in severe valgus [31], and increase the likelihood of procedural success in the event of revision surgery [32]. The option to modify initial cuts to achieve the desired plane of resection may lead surgeons to initially perform a more conservative resection to preserve bone stock, knowing that the feedback offered by navigation can help make postresection alterations if necessary. While this is one potential way in which resection verification and a better understanding of cutting errors could work to optimize TKA procedures, quantifying how cut verification alters resection procedure is outside the scope of this study; however, it but may be a potential avenue for future investigation.

The present study is not without limitations. Given the study design, we were unable to quantify what degree of cut variation may alter bone resection as a result of the real-time intraoperative feedback. Also, we did not evaluate component position on postoperative radiographs, which limited the ability to determine the final implant alignment, or to validate the feedback provided by the CAS. In this study, we sought only to quantify the magnitude of cutting errors that may occur during the procedure due to variables such as saw blade thickness, jig slot width, and technical variance. Additionally, although a power analysis shows that our sample size is akin to and consistent with similar studies, including Chua et al. and Plaskos et al., a larger sample size may improve the robustness and generalizability of our results [11, 15]. Future studies validating the feedback and assessing how this might alter the resections made during the TKA procedure would help better determine the clinical benefit of this feature.

## Conclusion

Quantifying the difference between planned and actual resections in primary TKA procedures using an imageless navigation device demonstrated that small but significant deviations in resection occur. Unnoticed or uncorrected, these deviations may lead to component malalignment and deleterious effect on implant survivorship and patient outcomes. The use of intraoperative navigation offers the ability to verify cut angles to minimize resection errors, which may offer a viable path to optimizing component placement. Ultimately, improving component alignment by ensuring optimal cuts are achieved will improve long-term success of knee replacement and postoperative knee functional outcomes.

## Data Availability

Data sharing not applicable to this article as no datasets were generated or analysed during the current study.
